# Predisposing factors and neonatal outcomes for twin-twin transfusion syndrome cases developing transient donor hydrops after fetoscopic laser coagulation: a case control study

**DOI:** 10.1186/s12884-019-2236-4

**Published:** 2019-03-11

**Authors:** Yao-Lung Chang, An-Shine Chao, Shuenn-Dyh Chang, Wen-Fang Li, Po-Jen Cheng

**Affiliations:** 1 Department of Obstetrics and Gynecology, Chang Gung Memorial Hospital, Chang Gung University College of Medicine, Tao-Yuan, Taiwan; 2 Department of Obstetrics and Gynecology, Chang Gung Memorial Hospital, Linkou Medical Center, 5, Fu-Shin Street, Kweishan, Taoyuan, Taiwan, Republic of China 333

**Keywords:** Twin-twin transfusion syndrome, Donor hydrops, Laser therapy, Quintero stage

## Abstract

**Background:**

Transient donor hydrops (TDH) is defined as donor hydrops developed within days after laser therapy for twin–twin transfusion syndrome (TTTS) followed by resolution later. The purpose of this study was to evaluate the incidence, neonatal outcomes and predisposing factors of post laser therapy TDH in severe TTTS.

**Methods:**

A total of 142 patients with severe TTTS who received laser therapy were included into this study. The pre-operative characteristics and neonatal outcomes were compared between TTTS with and without post laser therapy TDH. All live neonates received cranial ultrasound examination after delivery, mild cerebral injury was defined as exhibiting at least one of the following: intraventricular hemorrhage (IVH) grade I and II, lenticulostriate vasculopathy and subependymal pseudocysts; severe cerebral injury comprised at least one among the following: IVH grade III or grade IV, cystic periventriculoleukomalacia (PVL) grade II or more, porencephalic cysts, and ventricular dilatation. Fetal survival was defined as living more than 30 days after delivery.

**Results:**

Twenty four TTTS (16.9%) were found with TDH after laser therapy. Between TTTS with and without TDH after laser therapy, A total fetal survival rate of (77.1% (37/48) vs 72.9% (172/236), respectively, (*p* = 0.60) and a cerebral injury rate of 5.4% (2/37) vs 6.4% (11/172), respectively (*p* = 1.0)) were not significantly different. A less intertwin estimated fetal weight discordance (Odd ratio: 0.96 (95% confidence of interval (CI) as 0.90~0.99)) and a severe Quintero stage (defined as stage III and IV, Odd ratio: 3.92 (95% CI as 1.9~12.0)) were determined as the two predisposing factors in predicting the occurrence of donor TDH in TTTS after laser therapy.

## Conclusion

TDH after laser therapy in TTTS is not associated with poorer neonatal survival or more cerebral injury in surviving fetuses. The donor hemodynamic change caused by laser therapy is more prominent in TTTS with severe Quintero stages and less intertwin estimated fetal weight discordance before laser, thereby leading to a more increased risk of post laser TDH.

## Background

Twin–twin transfusion syndrome (TTTS) occurs in approximately 9% of monochorionic, diamniotic twin pregnancies [1]. Fetoscopic laser therapy is recognized as the first line therapy for stage II to IV TTTS diagnosed before 26 weeks of gestation [2], and though with some debate, also in stage I TTTS [3, 4]. The rationale of laser therapy for TTTS is to coagulate the placental vascular anastomoses in order to make the placenta become functionally dichorionic, resulting in the resolution of the polyhydramnios-oligohydramnios sequence. Coagulation of vascular anastomoses interrupts the imbalance of transfusion and consequently induces fetal hemodynamic changes that impose an impact on clinical presentations observable by ultrasound [5]. Transient donor hydrops (TDH), defined as donor hydrops within days after laser therapy for TTTS followed by resolution later, has been reported since1998 [6]. The etiology of TDH had been speculated to be a transient congestive heart failure associated with sudden interruption of intertwin transfusion, and its occurrence was reported unrelated to poorer fetal outcomes in a 40 case series (with 10 cases of TDH after laser therapy), where a less difference in intertwin weight discordance before laser therapy was found as a predisposing factor for TDH after laser therapy in TTTS [5]. Since there is a lack of further literature about the incidence, predisposing factors and fetal outcomes associate with TDH in TTTS post laser therapy. We herein perform an analysis of our consecutive cases of TTTS receiving laser therapy in order to find out the association of TDH with the fetal outcomes and the factors predisposing to the happening of TDH after laser therapy.

## Methods

The present study was a retrospective design, which was performed at Chang Gung Memorial Hospital, Taoyuan, Taiwan. The study was approved by the Institutional Review Board of the Chang Gung Medical Foundation (IRB # 201801353B0). The diagnosis of TTTS was based on the ultrasound findings defined by Quintero et al. [7]: monochorionic twin pregnancy and the presence of polyhydramnios in the recipient’s gestational sac (maximum vertical pocket > 8 cm) and oligohydramnios in the donor’s gestational sac (< 2 cm); with reverse TTTS was diagnosed with detection of polyhydramnios in the previous donor’s gestational sac and oligohydramnios in the previous recipient’s gestational sac after laser therapy. From October 2005 to April 2018, 150 patients with TTTS who received fetoscopic laser therapy at our hospital were included in this study; they were all given 50 mg of indomethacin as a tocolytic and prophylactic antibiotic therapy with 2 g Cefazolin as prophylactic antibiotic about one hour and 30 min before operation, respectively. Surgery was performed in the operating room under regional or local anesthesia with methods of selective laser photocoagulation of communicating vessels (SLPCV) [8] with or without Solomon technique [9]. The procedure was performed with a 2-mm 0-degree fetoscope (Storz 26,008 AA; Karl Storz GmbH, Tuttlingen, Germany) or a 3.5-mm 30-degree fetoscope (Storz 26,008 BUA, Karl Storz GmbH, Tuttlingen, Germany) for mainly anterior placenta; they carried a working channel on top for a 0.6 mm laser fiber; the anastomoses were selectively coagulated with 10–30 W Diode laser continue beams, according to the diameter of the targeted vessel; cefazolin 500 mg per six hours was given for 24 h after operation and nifedipine was the choice of tocolytic agent if necessary.

TTTS patients were followed up with serial ultrasound one day before, and every day after laser therapy if patient was admitted. All TTTS with TDH after laser therapy were subject to admission till TDH resolved.

Cases with fetal demise during the first 120 h after laser therapy or reverse TTTS found after operation were excluded from this study.

Donor hydrops was recognized as the presence of two or more following signs in the donor fetus: pleural effusion, ascites, pericardial effusion or skin edema. TDH in this study was defined as donor hydrops that happened within days after laser therapy for TTTS and resolved days after. Ductus venosus (DV) and umbilical artery (UA) Doppler of donor twins were evaluated at the time when TDH was diagnosed. UA blood flow velocity waveforms were obtained from a free-floating loop of the umbilical cord and the angle of insonation was maintained as close to 0° as possible. DV Doppler was depicted by using a two-dimensional and color Doppler imaging in a mid-sagittal section or oblique transection of the fetal abdomen. The sample volume was placed in the distal smallest portion of the vessel in order to record the highest blood velocities. The angle between the ultrasound beam and the direction of the blood flow was maintained as low as possible. Abnormal UA Doppler was defined as absence or reverse of end-diastolic flow (UA-AREDF) and abnormal UV Doppler was defined as absence or reverse of A wave. The preoperative characteristics, including maternal age, Quintero staging, gestational age at operation, estimated fetal weight, intertwin estimated fetal weight discordance (EFWD) and maximum vertical pocket (MVP) values of both fetuses were assessed and documented in the TDH and non-TDH groups. Severe Quintero stage was defined as stage III or IV TTTS. Estimated fetal weight one day before laser therapy was recorded and intertwin EFWD was calculated as “(**(**estimated fetal weight of recipient-estimated fetal weight of donor)/estimated fetal weight of recipient) X 100%)”, and birth weight discordance after delivery was calculated similarly. The outcomes of fetoscopic laser therapy for TTTS, including fetal survival (defined as living more than 30 days after delivery), gestational age at delivery, and interval between laser therapy and delivery were compared between the two groups. All live neonates received cranial ultrasound examination within one month after delivery; if two or more cranial ultrasound tests were performed, then the last examination result was selected. Mild cerebral injury was defined as having lesions detected by cranial ultrasound scans with the presence of at least one of the following: intraventricular hemorrhage (IVH) grade I and II, lenticulostriate vasculopathy and subependymal pseudocysts, while severe cerebral injury was regarded as presenting one of the following signs including IVH grade III or grade IV, cystic periventriculoleukomalacia (PVL) grade II or more, porencephalic cysts, and ventricular dilatation; ventricular dilatation was said to be present when the width of unilateral or both lateral ventricles exceeded the 97th percentile.

Statistical analysis was conducted using SPSS (version 11.0 for Windows; SPSS Inc., Chicago, IL, USA). Data are expressed as mean ± standard deviation, median [inter-quartile range] and frequency (%) as and when appropriate. Qualitative data were compared by means of X2 test or Fisher exact test. Continuous variables were tested for normality. Two-sample Student t test or Mann-Whitney U test was used to compare between groups for the continuous variable. A probability value of less than 0.05 was considered statistically significant. Logistic regression (forward stepwise mode) was chosen in order to find out the predisposing factors of TDH in TTTS after laser therapy. MVP of recipient, gestational ages of laser therapy and delivery, EFWD, birth weight of donor and recipient and severe Quintero stage were picked as variables that were selected to enter the mode when a probability value of less than 0.05 and removed when a probability value of more than 0.1. Odd ratios of variables were expressed as odds (95% confidence of interval (CI)).

## Result

During the study period, 150 TTTS received fetoscopic laser therapy at our hospital. During the first 120 h after laser therapy, six donors died in utero (with their three recipient counterparts surviving and three pairs of TTTS losing both fetuses in uterus). In one (16.7%) of the six cases, the donor showed hydrops in the second day after laser therapy and soon succumbed with the recipient surviving to delivery at gestational age of 27 weeks. The six TTTS were not included in further analysis. There were two cases presenting with donor hydrops after laser therapy but eventually developing reverse TTTS, which were also excluded. After exclusion of the 8 cases (5.3%) described above, overall there were 142 TTTS undergoing study and analysis. Among them there were 24 cases (16.9%) with TDH after laser therapy. Clinical features of the 142 cases are summarized in Table 1.

**Table 1:** Characteristics of twin-twin transfusion syndrome (TTTS) patients with and without transient donor hydrops (TDH) after laser therapy: data are expressed as mean ± standard deviation, median [inter-quartile range] and frequency (%)

	With TDH after laser therapy (*n* = 24)	Without TDH after laser therapy (*n* = 118)	*p*
Maternal age at laser therapy (year-old)	33.2 ± 5.1	31.4 ± 4.2	0.077*
Gestational age at operation (weeks)	20.7 ± 2.9	20.8 ± 2.6	0.89*
Amniotic fluid maximum vertical pocket (cm) of recipient twin	12.0 [6]	10.8 [3.69]	0.51#
Gestational age at delivery (weeks)	34.0 [6.0]	34.4 [5.1]	0.93#
Birth weight of liveborn recipients(g)	2180 [1312]	2010 [775]	0.97#
Birth weight of liveborn donors (g)	1955 [1330]	1635 [865]	0.20#
Severe Quintero stage (stage III and IV TTTS)	66.6% (16/24)	42.4% (50/118)	0.030※
Interval form laser therapy to delivery (days)	88 [57]	9 [40]	0.51#
Intertwin EFWD (%)	17.4 ± 12.8	24.7 ± 16.9	0.046*
Intertwin BWD (%)	− 0.6 [16.2] (*n* = 15)	16.5 [21.5] (*n* = 71)	< 0.001#
Donor survival	79.2% (19/24)	68.6% (81/118)	0.34※
Recipient survival	75.0% (18/24)	78.8% (93/118)	0.79※
Two fetuses survival rate	62.5% (15/24)	60.2% (71/118)	0.82※
Total survival	77.1% (37/48)	72.9% (172/236)	0.60※

*: Student t test

#: Mann-Whitney U test

※: Chi-square or Fisher’s exact test

*EFWD*: Estimated fetal weight discordance

*BWD*: birth weight discordance

EFWD was calculated as “((estimated fetal weight of recipient-estimated fetal weight of donor)/estimated fetal weight of recipient) X 100%)”

BWD was calculated as “((birth weight of recipient- birth weight weight of donor)/birth weight of recipient) X 100%)”

The mean gestational age at operation in all TTTS was 20.8 ± 2.6 weeks. Of the patients, 25 were at Quintero stage I (17.6%), 51 at stage II (35.9%), 46 at stage III (32.4%), and 20 were at stage IV (14.1%). The mean gestational age at delivery was 31.9 ± 5.3 weeks, total fetal survival rate 73.6% (209/284), at least single survival rate 86.6% (123/142) and dual survival rate was 60.6% (86/142). The mean time of TDH found in the 24 cases started 2.2 ± 1.2 days and resolved 7.2 ± 2.5 days after laser therapy. The mean duration of TDH in the 24 TTTS was 4.6 ± 2.4 days.

In the two cases where the donor hydrops failed to resolve spontaneously, their recipient twins eventually developed oligohydramnios due to reverse TTTS: among them, one received a repeat laser therapy (first laser at gestational age of 20 weeks, donor developing hydrops 3 weeks later and second laser therapy done at gestational age of 24 weeks) and delivered at 36 weeks resulting in two normal fetuses, and another case, undergoing laser therapy at 22 weeks with donor hydrops found 14 days later, delivered at 26 weeks with donor neonatal death (recipient survival). There was one case with TDH developing maternal mirror syndrome, the TDH found two days after laser therapy and eventually two live babies were delivered at gestational age of 36 weeks [10].

The two groups of TTTS with and without TDH after laser therapy did not differ significantly in the maternal age, gestational age at operation and MVP of recipient twins. TTTS cases with post laser TDH was found with less intertwin EFWD and a higher ratio of severe Quintero staging before laser therapy than without post laser TDH. After laser therapy, the gestational age of delivery was not significantly different between the two groups of TTTS. TTTS with TDH after laser therapy had less intertwin birth weight discordance in cases of two fetal survivals. Fetal survivals including two fetuses survival and total survival rates showed no significant difference between the two groups of TTTS (Table 1).

In the 24 cases with TDH after laser therapy, there were nine donor twins whose UA Doppler was abnormal before laser therapy and five (55.6%) of the nine donors the UA Doppler became normal when TDH was diagnosed. In the 15 donor twins with normal UA Doppler before operation, one donor (6.7%) showed abnormal UA Doppler found when TDH was diagnosed, the pair eventually aborted due to premature rupture of membranes at 16 days after laser therapy. All the 24 donor twins with TDH after laser therapy exhibited no abnormal DV Doppler changes, neither with absence, nor reverse of a wave when TDH was found.

In the 202 live babies that survived for more than 30 days, there were 13 cases (6.4%) diagnosed with brain injury by cranial ultrasound, among them five (2.4%) cases being severe brain injury. The incidences of mild, severe or total brain injury rate of live births were not significantly different between TTTS with and without TDH after laser therapy (Table 2).

**Table 2:** live born neonatal brain injury detected by ultrasound between twin-twin transfusion syndrome (TTTS) patients with and without transient donor hydrops (TDH) after laser therapy

	With TDH after laser therapy (*n* = 37)	Without TDH after laser therapy (*n* = 172)	*p*
Mild brain injury	1 (2.7%)	8 (4.7%)	1.0
Severe brain injury	1 (2.7%)	4 (2.3%)	1.0
Total brain injury	2 (5.4%)	11 (6.4%)	1.0

Mild cerebral injury was defined as having lesions detected by cranial ultrasound scans with the presence of at least one of the following: intraventricular hemorrhage (IVH) grade I and II, lenticulostriate vasculopathy and subependymal pseudocysts

Severe cerebral injury was defined as having at least one of the following: IVH grade III or grade IV, cystic periventriculoleukomalacia (PVL) grade II or more, porencephalic cysts, and ventricular dilatation. Ventricular dilatation was said to be present when the width of unilateral or both lateral ventricles exceeded the 97th percentile

*P* values were generated by fisher’s exact test

Logistic regression analysis variables showed less intertwin EFWD (Odd ratio: 0.96 (0.90~0.99)) and severe Quintero stage (Odd ratio: 3.9 (1.9~12.0)) as two predisposing factors to predict the donor TDH after laser therapy in TTTS.

## Discussion

This study revealed the incidence of TDH in TTTS after laser therapy is 16.9%, and its occurrence is not associated with fetal survival rate and brain injury rates in surviving neonates of TTTS after laser therapy. Before laser therapy, a combination of less intertwin EFWD and severe Quintero stage could predict the occurrence of TDH in TTTS after laser therapy.

Because the onset of TDH after laser therapy in this series was one to five days after operation, so we excluded cases of donor intrauterine death within five days after laser therapy in order to eliminate the effect of fetal death affecting the incidence of TDH. The range of resolution of TDH of this series was five to 14 days after laser therapy, with the longest duration of TDH lasting 10 days, which was compatible with a previous report that said TDH after laser therapy in TTTS usually appeared early after laser therapy (a mean of 2.2 days in this series) and persisted for a few days (a mean of 7.2 days in this series) [5].

The TDH after laser therapy in TTTS is thought happening due to a hypervolemic status after cessation of intertwin transfusion [5]. A less intertwin EFWD had been found in TTTS with TDH after laser therapy but the mechanism is still not clear [5]. The fetal weight discordance had been reported as correlated with the placenta territory discordance in monochorionic twin [11], so a reduced intertwin EFWD in monochorionic twin may indicate less placenta share discordance. An adequate individual placenta territory shared by donor twin in TTTS could predict an improved donor umbilical artery Doppler pattern after laser therapy [12]. Authors suspect the hemodynamic change in donor twin induced by laser therapy may be more prominent in cases where the placenta territory share in the donor twin was not severely low as indicated by a less intertwin EFWD. This may explain why the incidence of TDH after laser therapy in TTTS is increased in TTTS where the intertwin EFWD was not too prominent.

Laser therapy causing hemodynamic variations in TTTS had been reported mainly a manifestation in stages III–IV TTTS [13]. Quintero stage III and IV donors showed a significant increase in the umbilical blood flow and DV pulstility index (PI) and a reduction in the UA PI after laser therapy, but these findings were not observed in Quintero stage I and II TTTS [13]. Our finding that severe Quintero stage could predict the occurrence of TDH after laser therapy was consistent with what was previously thought that the donor twin hemodynamic variations after laser therapy was mainly manifested in stages III–IV TTTS [13]. Certainly not all TTTS with donor hydrops found after laser therapy present with favorable outcomes. As was described, there were two cases where present prolonged donor hydrops and reverse TTTS happened, and they were excluded from this study. The mean days of TDH found after laser therapy in our series was 2.2 days, and in the two cases of reverse TTTS, the initial donor hydrops found were 21 and 14 days after laser therapy. For reverse TTTS, it had ever been discovered that initial donor polyhydramnios appeared at 7 weeks [14] and 7 days [15] after laser therapy, but no donor hydrops had been reported. Authors suspect that donor hydrops found late after laser therapy could be a similar finding associated with reverse TTTS.

Previously we had reported that the severe neurodevelopment disability in live babies after laser therapy in TTTS was 6.7% in one year after delivery by combining brain magnetic resonance imaging (MRI) and neurodevelopmental assessment examinations [10]. In this study, with the limitation of using only cranial ultrasound to detect brain injury, the severe brain injury rate was 2.4%, which could be under-estimated if further examination like MRI or neurodevelopmental assessment was added on. But owing to no significant difference in minor and major brain injury rates between the survivors with and without TDH by cranial ultrasound examinations, authors suppose the TDH happening after laser therapy is not a significant contributing factor of brain injure in TTTS survivors.

There was however, a limitation in this study: we usually keep TTTS patients hospitalized for 3 days after laser therapy to observe if complications like premature labor, premature rupture of membranes or detachment of occur, during which period, the ultrasound exam was performed at least once per day; in cases where he donor was found only with skin or scalp edema but not fulfilling the diagnostic criteria of TDH, we would keep them admitted to observe if TDH would happen or not; all TTTS patients post laser therapy would be followed up at an weekly interval in the first three weeks after operation, then followed up with a bi-weekly interval if condition was stable; as a result, we might miss out on cases of TDH appearing between 4~11 days after laser therapy.

## Conclusion

TDH after laser therapy in TTTS is not associated with poorer fetal survival rate or increased neonatal brain injury rate in surviving fetuses. Due to more severe hemodynamic change in donor twin after laser therapy in cases of TTTS with a less intertwin EFWD and severe Quintero stage, hence the possibility of TDH after laser therapy increases.

## Abbreviations

BWD: Birth weight discordance
CI: Confidence of interval
EFWD: Estimated fetal weight discordance
IVH: Intraventricular hemorrhage
MRI: Magnetic resonance imaging
MVP: Maximum vertical pocket
PI: Pulstility index
PVL: Periventriculoleukomalacia
SLPCV: Selective laser photocoagulation of communicating vessels
TDH: Transient donor hydrops
TTTS: Twin–twin transfusion syndrome
